# Tandem duplication underlies diversification of the gymnosperm-specific TPS-d subfamily

**DOI:** 10.1093/hr/uhag195

**Published:** 2026-05-20

**Authors:** Yuwei Liang, Xin Geng, Yijun Shen, Kuang Sheng, Xinyi Zhou, Danqing Li, Xiuyun Wang, Chuan Dong, Xiaofan Zhou, Liangsheng Zhang, Jiasheng Wu, Chengjun Zhang

**Affiliations:** State Key Laboratory for Development and Utilization of Forest Food Resources, Zhejiang A&F University, Hangzhou, China; Zhejiang Key Laboratory of Non-wood Forest Products Quality Regulation and Processing Utilization, Zhejiang A&F University, Hangzhou, China; State Key Laboratory for Development and Utilization of Forest Food Resources, Zhejiang A&F University, Hangzhou, China; Zhejiang Key Laboratory of Non-wood Forest Products Quality Regulation and Processing Utilization, Zhejiang A&F University, Hangzhou, China; State Key Laboratory for Development and Utilization of Forest Food Resources, Zhejiang A&F University, Hangzhou, China; Zhejiang Key Laboratory of Non-wood Forest Products Quality Regulation and Processing Utilization, Zhejiang A&F University, Hangzhou, China; Biotechnology and Genetic Germplasm Resource Research Institute, Yunnan Academy of Agricultural Sciences, Key Lab of Agricultural Biotechnology of Yunnan, Kunming, China; Zhejiang Key Laboratory of Horticultural Crop Quality Improvement, Zhejiang University, Hangzhou, China; Laboratory of Key Trait Regulation and Germplasm Innovation in Bulbous and Perennial Herbaceous Flowers, School of Civil Engineering and Architecture, Zhejiang Sci-Tech University, Hangzhou, China; Genomics and Genetic Engineering Laboratory of Ornamental Plants, College of Agriculture and Biotechnology, Zhejiang University, Hangzhou, China; State Key Laboratory for Development and Utilization of Forest Food Resources, Zhejiang A&F University, Hangzhou, China; Zhejiang Key Laboratory of Non-wood Forest Products Quality Regulation and Processing Utilization, Zhejiang A&F University, Hangzhou, China; State Key Laboratory for Conservation and Utilization of Subtropical Agro-Bioresources, Guangdong Laboratory for Lingnan Modern Agriculture, Guangdong Province Key Laboratory of Microbial Signals and Disease Control, Integrative Microbiology Research Center, South China Agricultural University, Guangzhou, China; Zhejiang Key Laboratory of Horticultural Crop Quality Improvement, Zhejiang University, Hangzhou, China; State Key Laboratory for Development and Utilization of Forest Food Resources, Zhejiang A&F University, Hangzhou, China; Zhejiang Key Laboratory of Non-wood Forest Products Quality Regulation and Processing Utilization, Zhejiang A&F University, Hangzhou, China; State Key Laboratory for Development and Utilization of Forest Food Resources, Zhejiang A&F University, Hangzhou, China; Zhejiang Key Laboratory of Non-wood Forest Products Quality Regulation and Processing Utilization, Zhejiang A&F University, Hangzhou, China

## Abstract

Terpene synthases (TPSs) are key drivers of terpenoid diversity in plants, yet the diversity and mechanisms driving their diversification in gymnosperms remain poorly understood. Through comparative genomic analyses of 16 representative land plant species, we demonstrate that tandem duplication (TD) plays an important role in the expansion of gymnosperm-specific TPS-d subfamily. These TD-generated TPS-d genes exhibit elevated sequence variability and markedly increased tissue specificity (τ) compared with conserved primary-metabolic TPS-c and TPS-e/f genes, indicating rapid functional and regulatory divergence following duplication. Together, our results propose a comprehensive evolutionary framework that illustrates how TD, sequence divergence, and expression diversification collectively shape TPS-d gene evolution in gymnosperms, providing new insights into lineage-specific terpenoid profiles and ecological adaptation in gymnosperms.

## Introduction

Primary metabolism is generally considered highly conserved across land plants, encompassing fundamental processes such as carbohydrate, amino acid, and organic acid metabolism that rely on photosynthetic carbon fixation for energy and biomass production [1]. In contrast, the emergence and diversification of specialized metabolism represent key evolutionary innovations that accompanied plant colonization of terrestrial environments, enabling adaptation to diverse ecological challenges [1, 2]. This metabolic diversification has resulted in the production of several hundred thousand structurally distinct specialized metabolites, with the actual number likely far exceeding current estimates [3]. Among specialized metabolites, terpenoids constitute one of the largest and most structurally diverse classes of natural products [4–7]. Although terpenoids are produced by organisms across all domains of life, green plants, particularly angiosperms, exhibit exceptional diversity in terpenoid varieties and abundance, underpinning a wide range of ecological functions and applications [8]. While certain terpenoids, such as gibberellins, function as essential phytohormones in primary metabolism, the majority act as specialized metabolites involved in defense, communication, and environmental interactions [6, 9]. Beyond their ecological roles, plant terpenoids also hold considerable economic values as pharmaceuticals, fragrances, industrial materials (e.g. diterpene resin acids, abbreviated as DRAs), and potential biofuel precursors [10–13].

The extraordinary structural diversity of terpenoids is largely generated by terpene synthases (TPSs), which catalyze the conversion of universal C5 isoprenoid precursors into diverse hydrocarbon scaffolds [5, 7]. These scaffolds are subsequently modified by tailoring enzymes, particularly cytochrome P450 monooxygenases, to yield the final array of bioactive compounds [14–16]. Plant TPSs comprise a moderately sized multigene family, typically 30–100 members per genome, and are classified into seven subfamilies (TPS-a, -b, -c, -d, -e/f, -g, and -h) based on phylogenetic relationships [17, 18]. Among these, TPS-c and TPS-e/f genes are deeply conserved across seed plants and participate in primary metabolism, whereas TPSs involved in specialized metabolism show strong lineage-specific diversification [17, 18].

Gymnosperms provide a particularly informative system for investigating the evolution of specialized terpenoid metabolism [19]. They possess a unique TPS-d subfamily that is absent from flowering plants and is further subdivided into TPS-d1, TPS-d2, and TPS-d3 based on structural and catalytic characteristics [17, 20]. TPS-d enzymes generate complex mixtures of mono-, sesqui-, and diterpenoids that accumulate in volatile emissions and oleoresin secretions, allowing them to cope with attacks from pests and pathogens [18, 21]. Notably, DRAs, such as abietic acid, are core defensive compounds in conifer resins. These DRAs are widely distributed across roots, stems, leaves, cones, and seed tissues in conifers and are primarily synthesized by diterpene synthases (di-TPSs) belonging to the TPS-d3 subfamily [20], which play a central role in resistance to herbivores and pathogens [18, 22]. Functional validation has been conducted for a broad spectrum of TPS-d3 genes associated DRA biosynthesis [23–25]. Notably, TPS-d3 genes encoding taxadiene synthase, an indispensable enzyme in paclitaxel biosynthesis, have been subjected to functional characterization in numerous *Taxus* species [26, 27]. Beyond TPS-d3 genes, functional identification and validation have also been achieved for TPS-d1 subfamily members that mainly produce a diversity of monoterpene synthases (e.g. limonene synthase, pinene synthase, and terpinolene synthase, etc.) [25, 28], as well as TPS-d2 members encoding sesquiterpene synthases (e.g. longifolene synthase, farnesol synthase, and humulene synthase, etc.) [20, 25].

Despite their ecological and evolutionary importance, both TPS-d diversity and mechanisms underlying its diversification in gymnosperms remain poorly understood. Previous studies have been limited by the scarcity of high-quality gymnosperm genomes, owing to their exceptionally large and complex genome architectures [29–32], which has limited the comprehensive identification and comparative analyses of TPS genes. Approximately a decade ago, the understanding of the quantity, structural and functional complexity, and phylogeny of TPSs in gymnosperms was solely based on the targeted cDNA cloning and characterization of conifer species as well as research on a small number of TPS in other gymnosperms [20, 23–25, 33, 34]. To date, functions of at least 112 TPS genes are experimentally characterized across all conifers [20]. However, most gymnosperm TPSs that possess clear biochemical functions are derived from the Pinaceae family. Consequently, both the diversity and evolutionary trajectories of TPSs, particularly TPS-d members, in other gymnosperm families, such as Taxaceae, remain largely unexplored.

As a result, it remains unclear how TPS-d genes expanded, diversified, and acquired specialized functions across gymnosperm lineages. To address this gap, we conducted a comprehensive evolutionary analysis of TPS genes using 10 chromosome-level gymnosperm genomes together with six other representative land plant genomes from other major lineages. This study is expected to advance our understanding of TPS-d diversity across three key dimensions: a comprehensive characterization of the TPS-d gene repertoire in gymnosperms, as well as the dissection of its diversification patterns at the levels of copy number, sequence variation, and expression dynamics (i); in-depth elucidation of the molecular mechanisms underpinning TPS-d diversification (ii); insight into the contribution of TPS-d diversity to the structural and functional diversity of terpenoids (iii).

## Result

### Genome-wide identification and phylogenomic characterization of TPS genes

To characterize the diversity and evolution of TPS genes, we performed a comparative phylogenomic analysis across 16 representative plant species from 11 orders, including one fern (*Alsophila spinulosa*), 10 gymnosperms, one basal angiosperm (*Nymphaea colorata*), two monocots (*Acorus gramineus* and *Oryza sativa*), and two dicots (*Vitis vinifera* and *Arabidopsis thaliana*) (Fig. 1A). All genomes included in this study have been assembled to chromosome levels (Supplementary Data 1). Most species display complete BUSCOs (C) (≥ 80%), confirming high-quality gene annotation completeness (Supplementary Data 1). Notably, despite their exceptionally large genome sizes, most of the gymnosperm assemblies exhibit high integrity, with scaffold N50 values exceeding 600 Mb (Supplementary Data 1).

**Figure 1:**
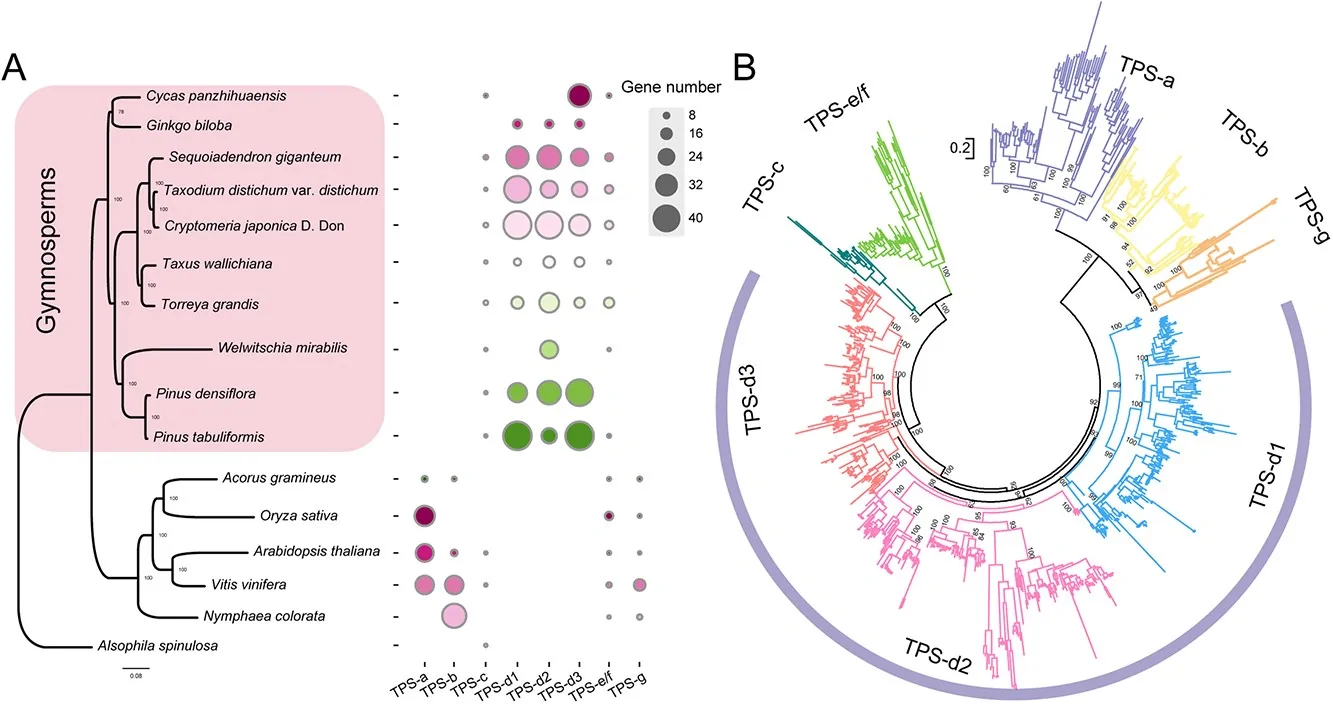
Phylogenetic relationships of 16 plant genomes and their terpene synthase (TPS) gene families. (A) Species phylogeny constructed using *Alsophila spinulosa* as the outgroup. (B) Maximum-likelihood gene tree of TPS family members across 16 species, highlighting the diversification of TPS subfamilies. Bootstraps of main clades were labeled.

The species-level phylogeny based on single copy orthologous genes obtained by Orthofinder was fully consistent with established relationships (Fig. 1A). Particularly within gymnosperms, *Ginkgo biloba* and *Cycas panzhihuaensis* represent the earliest-diverging gymnosperms, while Taxaceae and Taxodiaceae clustered together, and Welwitschiaceae was sister to Pinaceae (Fig. 1A).

Using Hidden Markov Models of Terpene_synth_C (PF03936) and Terpene_synth (PF01397), we identified a total of 903 putative TPS genes from these 16 land plant genomes (Fig. 1A). Across these studied species, TPS gene numbers exhibited a broad range of variations, spanning from just one gene in *A. spinulosa* to 120 genes in *Cryptomeria japonica* D. Don, with a mean TPS gene family size of 58 members (Fig. 1A). Phylogenetic analysis divided these TPS members into six subfamilies, namely TPS-a, TPS-b, TPS-g, TPS-e/f, TPS-c, and TPS-d (Fig. 1A and B). Whereas TPS-a, TPS-b, and TPS-g are angiosperm-specific clades, the TPS-d clade is exclusive to gymnosperms (Fig. 1A and B) [17]. The gymnosperm-specific TPS-d subfamily was further classified into three subclades, TPS-d1, TPS-d2, and TPS-d3, each containing ~200 genes (Fig. 1A and B). Most gymnosperms harbored all three TPS-d subclades, with an average of 65 TPS-d genes identified per species (Fig. 1A).

### Lineage-specific expansion of TPS-d genes through tandem duplication in gymnosperms

Phylogenetic reconstruction of the TPS-d subfamily revealed that TPS-d3 diverged earliest, followed by the separation of TPS-d1 and TPS-d2 (Fig. 1B). According to previous studies, TPS-d1 is primarily associated with monoterpene synthases, TPS-d2 with sesquiterpene synthases, and TPS-d3 with diterpene synthases [24, 25]. Notably, none of the three TPS-d subclades showed phylogenetic signatures of whole-genome duplication (e.g. symmetric sister subclades with similar species composition, Fig. 2A), suggesting that WGD is unlikely to be the primary driver of TPS-d expansion. Instead, TPS-d genes from the same species frequently clustered into species-specific subclades, with substantial variation in gene copy number among species, indicating lineage-specific expansions (Fig. 2A).

**Figure 2:**
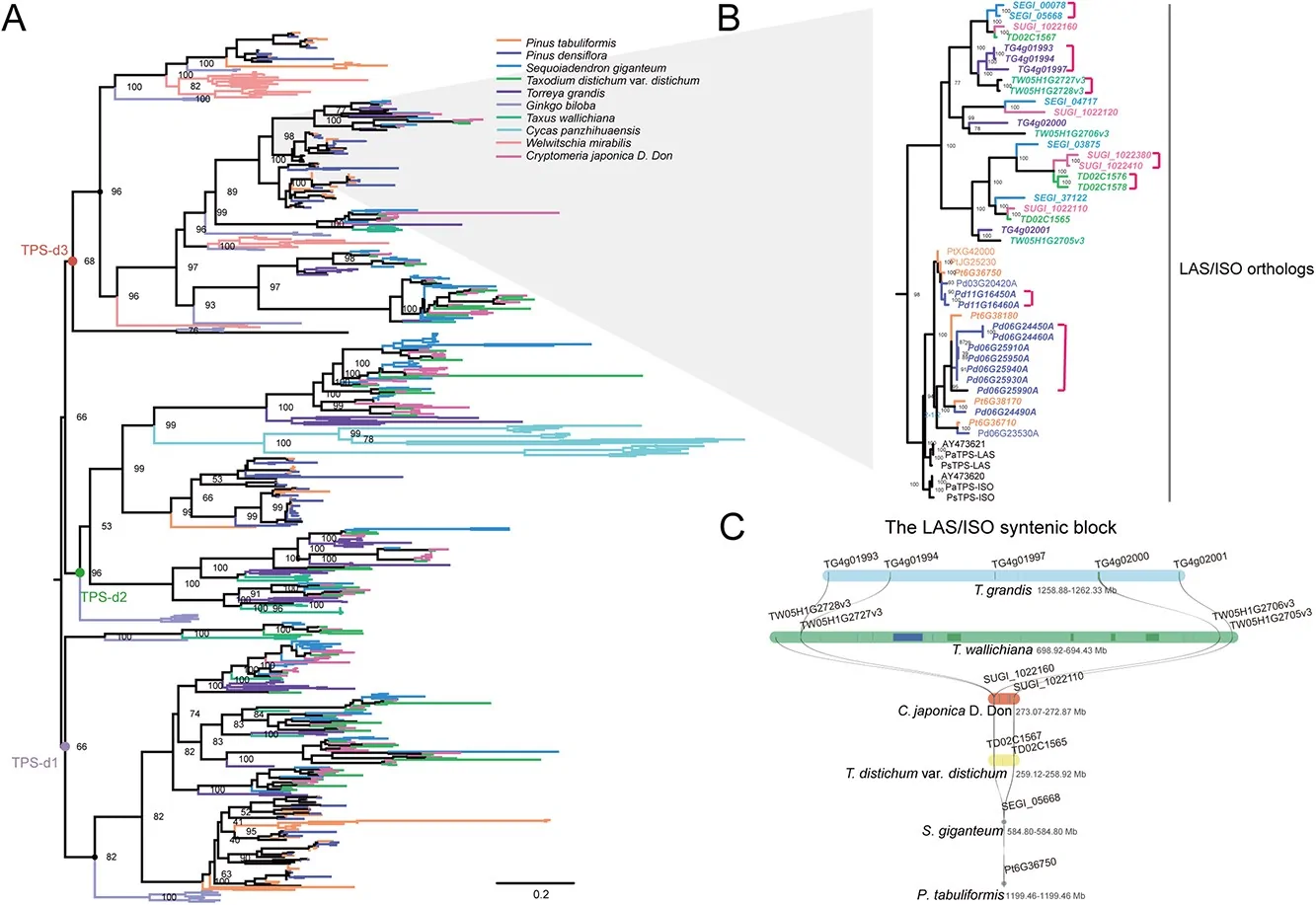
Evolutionary diversification and lineage-specific expansion of TPS-d genes in gymnosperms. (A) Phylogenetic reconstruction showing the diversification of the gymnosperm-specific TPS-d clade into ancestral (d3), and derived (d1 and d2) sub-lineages. (B) Detailed subtree illustrating orthologous relationships among LAS/ISO genes involved in diterpene resin acid biosynthesis. Genes generated by TD were represented in italic bold fonts, and species-specific tandem duplicates were enclosed in parentheses. Levopimaradiene/abietadiene synthase, LAS; isopimara-7,15-diene synthase, ISO. (C) Microsynteny analysis of the LAS/ISO genomic block across representative gymnosperms.

Based on gene positions along chromosomes, we found that 68% of TPS-d genes were in tandemly duplicated arrays, ranging from 50.00% (*W. mirabilis*) to 82.50% (*T. distichum* var. *distichum*), suggesting that TD, rather than WGD, has played a dominant role in TPS-d expansion (Table S1). The three TPS-d subclades (d1, d2, and d3) showed similar average numbers of TD-generated members, which accounted for ~62%–72% of each subclade (Table S1). One exception was *T. wallichiana*, where all TPS-d2 genes were TD-derived, compared to only 28.57% in TPS-d1 (Table S1)*.* Tandem arrays of TPS-d genes varied considerably in size, from small clusters of 2–3 genes to large blocks of over 10 adjacent genes (Fig. S1). Based on the *Ks* density distributions of tandemly duplicated TPS genes across different gymnosperm species, and assuming a synonymous substitution rate of *r* = 6.5 × 10^−9^ substitutions per site per year (*T* = *Ks*/2*r*) [35], we found that the majority of TD events in gymnosperms are concentrated within a lower *Ks* range (0–0.3), corresponding to ~0–23 Mya (Neogene) (Fig. S2, Supplementary Data 16). In addition, a secondary peak was detected in some species at higher *Ks* values (0.3–0.5), corresponding to ~23–38 Mya, which likely represented earlier duplication events during the Paleogene (Fig. S2). These observations highlight a clear temporal layering in the expansion of gymnosperm TPS families, with the Neogene as its dominant phase, indicating that most TD events occurred after the divergence of extant gymnosperm lineages (Fig. S2). Notably, this period coincides with major global climatic transitions, including progressive cooling and increased aridification during the Neogene, which may have imposed selective pressures favoring the expansion and diversification of TPS genes involved in stress response and defense [36]. Within these TPS tandem arrays, several are lineage-specific and comprise tandem duplicates that arose after lineage diversification. For instance, genes encoding levopimaradiene/abietadiene synthase (LAS) and isopimara-7,15-diene synthase (ISO), key enzymes in DRA biosynthesis, consistently formed lineage-specific tandem arrays (2–9 copies) across *P. tabuliformis*, *P. densiflora*, *T. grandis*, and other conifers (Fig. 2B and C, Fig. S1A). Within the LAS/ISO syntenic block (1258.88–262.33 Mb on chromosome 4 of *T. grandis*), *S. giganteum* and *P. tabuliformis* each retained one TPS gene, whereas five and four tandem TPS genes were present in this syntenic region in *T. grandis* and *T. wallichiana*, respectively (Fig. 2C). In *P. densiflora*, the genomic region harboring tandem duplicated LAS/ISO orthologs Pd06G25910A–Pd06G25990A showed no syntenic genes in other gymnosperms. By contrast, within the LAS/ISO syntenic block containing tandem duplicates Pd06G24450A–Pd06G24490A, only one syntenic gene (Pt6G38180) was detected in *P. tabuliformis* (Fig. S3). Additionally, these tandem duplicates formed a species-specific monophyletic clade (Fig. 2B). These findings suggest that following the divergence from *P. tabuliformis*, TD events occurred in *P. densiflora*, giving rise to these LAS/ISO copies. Consistently, *Ks* values for these *P. densiflora* tandem duplicates were lower than the *Ks* peak value (*Ks* ≈ 0.3) representing the divergence of *P. densiflora* from *P. tabuliformis*, further supporting recent, species-specific TD events (Fig. S4).

Together, these results strongly support that the diversification of TPS-d genes in gymnosperms might been predominantly driven by lineage-specific tandem duplications (TDs) occurring after species divergence, rather than by ancient WGD events.

### Sequence evolutionary dynamics among TPS subfamilies

Because amino acid substitutions in TPS genes can lead to substantial changes in catalytic activity and product profiles [37–39], we quantified amino acid polymorphisms (AAP) across TPS proteins from these 16 land plant species to understand how TD shaped TPS sequence evolution and thus functional diversification (Fig. 3A, Figs S5–S8, Table S2). AAP values ranged from 0.36 in *N*. *colorata* to 0.60 in *A. thaliana*, with an overall mean of 0.50, reflecting substantial sequence variability across the TPS family (Fig. 3A, Table S2). Distinct AAP patterns were observed across TPS subfamilies (Fig. 3A, Fig. S5). The TPS-c and TPS-e/f subfamilies involved in primary metabolism showed the lowest mean AAP values (0.19 and 0.27, respectively), consistent with their conserved functional roles (Fig. 3A). In contrast, subfamilies associated with specialized terpenoid metabolism exhibited substantially higher variability (Fig. 3A). For instance, average AAP values for the gymnosperm-specific TPS-d1, TPS-d2, and TPS-d3 subclades were 0.33, 0.35, and 0.37, respectively (Fig. S5). Additionally, TPS-d genes consistently exhibited higher AAP values than other TPS subfamilies within the same species, and these elevated AAP values were positively associated with TPS-d gene copy number (Fig. 3A–C, Fig. S8). This pattern was evident in both *C. japonica* and *T. grandis*, where TPS-d subclades exhibited markedly higher AAP values than the conserved TPS-c and TPS-e/f subfamilies (Fig. 3B and C). Within gymnosperms, relatively lower AAP values were detected in *G. biloba*, *P. tabuliformis*, and *P. densiflora* (Fig. 3A, Fig. S8).

**Figure 3:**
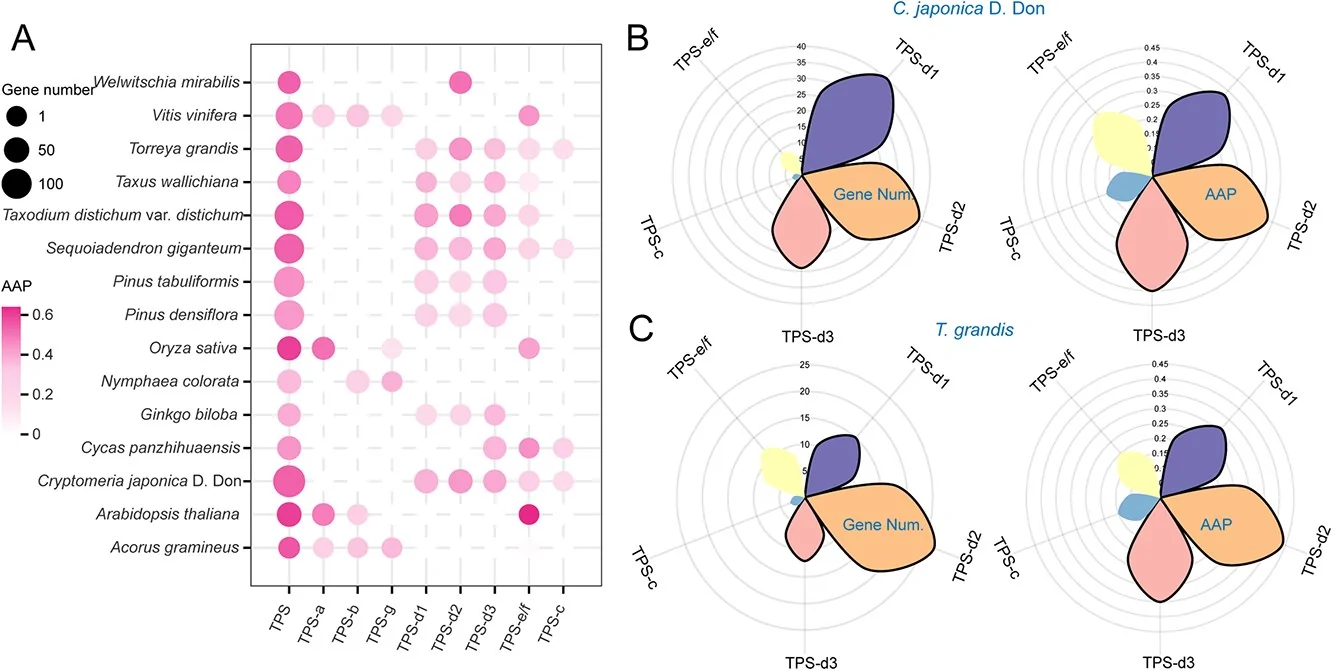
AAP and gene family size variation in TPS members across 16 plant genomes. (A) Heatmap showing TPS gene number and AAP values across species and subfamilies. The AAP values of TPS families/subfamilies were calculated across different species, and subfamilies containing only one TPS member were excluded from the calculation. (B) Radar plots illustrating AAP values of TPS subfamilies in *C. japonica* and *T. grandis*. Number, Num.

Subsequently, the AAP values of TD-generated TPS-d genes were calculated (Table S3). Overall, AAP values varied substantially among species and TPS-d subclades, ranging from 0.29 to 0.55, indicating heterogeneous levels of sequence divergence following TD (Table S3). Relatively low AAP values (≤0.30) of TD-generated TPS genes were observed in several *P. tabuliformis* and *G. biloba* TPS-d subclades, suggesting strong sequence conservation among duplicated copies (Table S3). In contrast, higher AAP values (>0.45) were detected in multiple lineages, e.g. *C. panzhihuaensis*, reflecting pronounced amino acid divergence among TD-derived TPS-d genes (Table S3). The highest AAP value was observed in the TPS-d2 subclade of *T. grandis* (AAP = 0.55) (Table S3).

To investigate whether amino acid variations among tandemly duplicated genes affect substrate binding, we performed protein structure prediction and molecular docking analysis for five representative LAS/ISO orthologous genes (TG4g01993, TG4g01994, TG4g01997, TG4g02000, and TG4g02001) (Fig. S9). All these five proteins exhibited highly similar overall three-dimensional structures, consistent with their origin from TD and indicating strong structural conservation at the global level. However, local differences were observed in the substrate-binding pockets (Fig. S9). Docking results revealed that the ligand (geranyl pyrophosphate) could be accommodated within a conserved catalytic cavity in all proteins, but the interacting residues and interaction patterns varied among paralogs (Fig. S9). For example, TG4g01994 showed interactions involving residues such as ARG and GLY, whereas TG4g01997 and TG4g02000 involved distinct residues (e.g. GLN, PHE, TRP, and GLU), suggesting divergence in the microenvironment of the binding pocket (Fig. S9). These differences were further reflected in the hydrogen bond distances and spatial orientations of the ligand (Fig. S9). The calculated binding energies ranged from −5.485 to −6.039 kcal/mol, with TG4g01994 exhibiting the strongest binding affinity (Fig. S9). Although the overall binding capacities were comparable, subtle differences in binding energy and residue interactions suggest potential functional divergence among these tandem duplicates. Taken together, these results indicate that while TD preserved the overall TPS protein fold, amino acid substitutions in key binding regions may contribute to fine-scale differentiation in substrate recognition or catalytic efficiency.

Collectively, these patterns indicate that TD is often followed by rapid protein sequence divergence in specific TPS-d subclades. Thus, TD may have played an important role in functional differentiation and contributed to metabolic innovation across gymnosperm lineages.

### Diverse expression patterns associated with TPS functional specialization

Functional divergence of TPS genes can be inferred from their distinct expression patterns [8, 39]. To infer the potential functional divergence of TPS family members, we characterized their gene expression across multiple tissues in representative species using publicly available RNA-seq datasets (Fig. 4, Fig. S10, Supplementary Data 2–15). Genes expressed ubiquitously were marked in blue boxes, while strongly tissue-specific genes were highlighted in yellow (Fig. 4). Genes with maximum transcripts per million (TPM) < 5 were excluded from downstream analysis. Consistent with their conserved biochemical roles, TPS-e/f members showed broad tissue expression, whereas TPS-d genes exhibited highly variable transcriptional patterns, including both specialized and ubiquitous profiles (Fig. 4, Fig. S10).

**Figure 4:**
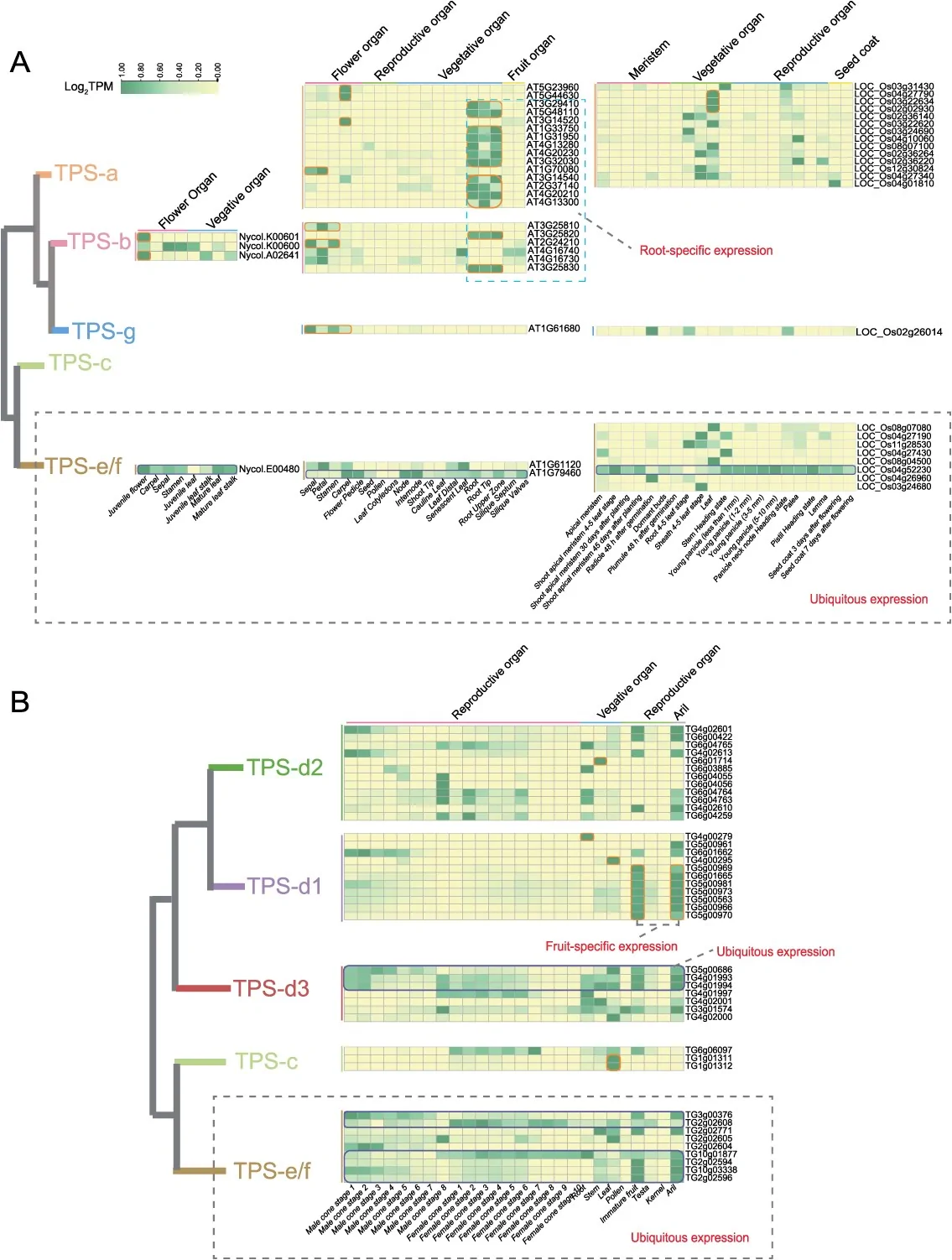
Tissue-specific and ubiquitous expression patterns of TPS genes across representative species. TPS genes with broad expression across tissues are highlighted in blue boxes, whereas strongly tissue-specific genes are highlighted in yellow boxes. Genes with maximum TPM < 5 were considered not significantly expressed and excluded from downstream analyses. LOC_Os04g52230 (*OsKS1*), LOC_Os04g27790 (*OsTPS24*), LOC_Os03a22634 (*OsTPS10*), AT1G79460 (*AtKS1*).

In the basal angiosperm *N. colorata*, only ~10% of TPS genes were expressed, all with strong floral specificity (Fig. 4A). Notably, Nycol.K00601 (TPS-b) was particularly enriched in juvenile floral tissues, suggesting involvement in floral scent or defense volatiles (Fig. 4A). In the dicot model *A. thaliana*, several TPS-a, TPS-b, and TPS-g genes exhibited organ-specific expression, whereas the TPS-e/f member AT1G79460 (*AtKS1*) was widely expressed (Fig. 4A). In rice (monocot), only the TPS-e/f gene LOC_Os04g52230 (*OsKS1*) was substantially expressed across multiple tissues, whereas three TPS-a genes (*OsTPS24*, *OsTPS10*, and LOC_Os02g02930) exhibited leaf-specific expression (Fig. 4A).

In gymnosperms, TPS-e/f genes exhibited both ubiquitous and tissue-specific expression patterns across *T. wallichiana*, *P. tabuliformis*, *C. japonica* D. Don, and *T. grandis* (Fig. 4B, Fig. S10). *T. grandis* exhibited a notably higher number of expressed TPS-e/f genes. Nine were broadly expressed across reproductive organs, immature fruits, and arils, while one was preferentially expressed in arils and immature fruits (Fig. 4B, Fig. S10). For TPS-d members, mixed ubiquitous and tissue-specific expression patterns were observed (Fig. 4, Fig. S10). Representative patterns were observed. *T. wallichiana* TPS-d1 genes expressed broadly across tissues, TPS-d2 genes enriched in bark and stem, and TPS-d3 genes highly expressed in epidermis and leaves (Fig. S10B). *P. tabuliformis* TPS-d genes highly expressed in male and female cones, with only four preferentially expressed in needles (Fig. S10F). And in *C. japonica* D. Don, TPS-d genes showed ubiquitous expression in shoots, leaves, and bark, with 13 significantly expressed in roots (Fig. S10G). In *T. grandis,* TPS-d3 genes exhibited a more ubiquitous expression pattern compared with TPS-d1 and d2 genes (Fig. 4). For instance, the LAS/ISO orthologs TG4g01993 and TG4g01994 were expressed across all tissues, consistent with the ubiquitous accumulation of DRAs in *T. grandis* and supporting their role in DRA biosynthesis (Fig. S11). Whereas TPS-d1 members displayed strong expression in immature kernels and arils, consistent with their contribution to monoterpene production in these tissues [40] (Fig. 4).

Together, these findings highlight distinct differences in expression range across TPS subfamilies. TPS-e/f genes are conserved and broadly functional, in contrast to dynamically regulated TPS-d genes that drive specialized terpenoid metabolism in gymnosperms.

### Expression specificity (τ) revealed functional divergence of TPS genes

To further investigate tissue-specific expression of TPS genes, we calculated τ values (ranging from 0 for ubiquitous expression to 1 for highly tissue-specific expression) across all examined species. Overall, τ values varied widely from <0.25 to >0.85, with a mean value of 0.77, indicating specialized transcriptional regulation across the family (Table S4).

Obvious differences in tissue specificity were observed among TPS subfamilies. The evolutionarily conserved TPS-e/f lineage displayed the lowest τ values (average τ = 0.67), consistent with its essential role in primary metabolism requiring broad expression (Figs S12–S16). In contrast, gymnosperm-specific TPS-d genes exhibited substantially higher τ values, with mean τ values of 0.87, 0.87, and 0.84 for TPS-d1, TPS-d2, and TPS-d3, respectively (Figs S12, S15, and  S16). We further evaluated τ values for tandem duplicated TPS-d genes across seven representative gymnosperms (Fig. S17). Mean τ values of these genes were consistently high (0.72–0.94), indicating that they generally undergo strong tissue-specific expression (Fig. S17). The highest mean τ was detected in *T. grandis*, followed by *G. biloba* and *C. panzhihuaensis*, suggesting particularly pronounced expression specialization in these species (Fig. S17). In contrast, relatively lower but still elevated τ values were detected in *T. wallichiana* and *P. tabuliformis*, implying a comparatively broader expression profile (Fig. S17). These results indicate that TD in TPS-d genes is commonly accompanied by rapid expression divergence.

We also identified dominant expression tissues as factor of major terpenoid biosynthesis sites in angiosperms, TPS genes were predominantly expressed in flowers, leaves, and roots (Fig. S18). A broadly similar pattern was observed in gymnosperms, although lineage-specific differences were also evident (Figs S19 and S20). For example, roots exhibited high TPS expressions in *C*. *japonica*, *C*. *panzhihuaensis*, and *T. grandis*, whereas in *W. mirabilis*, only a single TPS gene showed root-preferential expression (Figs S19 and S20). In addition, gymnosperm TPS genes were frequently highly expressed in needles and male and female cones, which are functionally analogous to leaves and flowers in angiosperms (Figs S19 and S20). These results indicate that, despite lineage-specific organ innovations, the major anatomical sites of terpenoid biosynthesis are broadly conserved between angiosperms and gymnosperms.

### Integrated analysis of TPS evolution, tissue-specific expression, and terpenoid accumulation in gymnosperms: A case study of *T. grandis*

*T*. *grandis* is an economically important tree species in the Taxaceae, which produces abundant terpenoids, making this species an ideal model for studying terpenoid metabolism [40]. To link tissue-specific terpenoid accumulation with TPS regulatory divergence, we integrated untargeted metabolomic and transcriptomic analyses across six tissues of *T. grandis* (Fig. S11). Untargeted metabolomic profiling revealed obvious differences in terpenoid composition and abundance among tissues (Fig. S11). Hierarchical clustering of terpenoid profiles demonstrated clear tissue-specific accumulation patterns, with distinct groups of metabolites preferentially enriched in reproductive, vegetative, and storage tissues, with kernels displaying a comparatively restricted terpenoid profile (Fig. S11).

Consistent with metabolic profiles, τ-based expression analysis revealed strong tissue-preferential expression of TPS genes (Figs S11 and S21). Kernels showed the lowest terpenoid content, in line with weak TPS expression in this tissue (Figs S11 and S21). By contrast, arils, roots, and male cones displayed the highest proportion of TPS genes with high τ values, consistent with their more abundant and diverse terpenoid profiles (Fig. 5A and Figs S11 and S21). TPS genes preferentially expressed in arils included both conserved TPS-e/f members and expanded TPS-d lineages (Fig. S21). Notably, TPS-e/f genes in *T. grandis* exhibited higher tissue specificity (mean τ = 0.83) than in other gymnosperms (Fig. S16D, Table S4). Other subfamilies in *T. grandis* showed even higher τ values, indicating strong expression specialization (Fig. S16D, Table S4). Together, these results indicate that terpenoid biosynthesis in *T. grandis* arils depends on the combined functions of conserved TPS-e/f genes and diversified TPS-d subfamilies.

**Figure 5:**
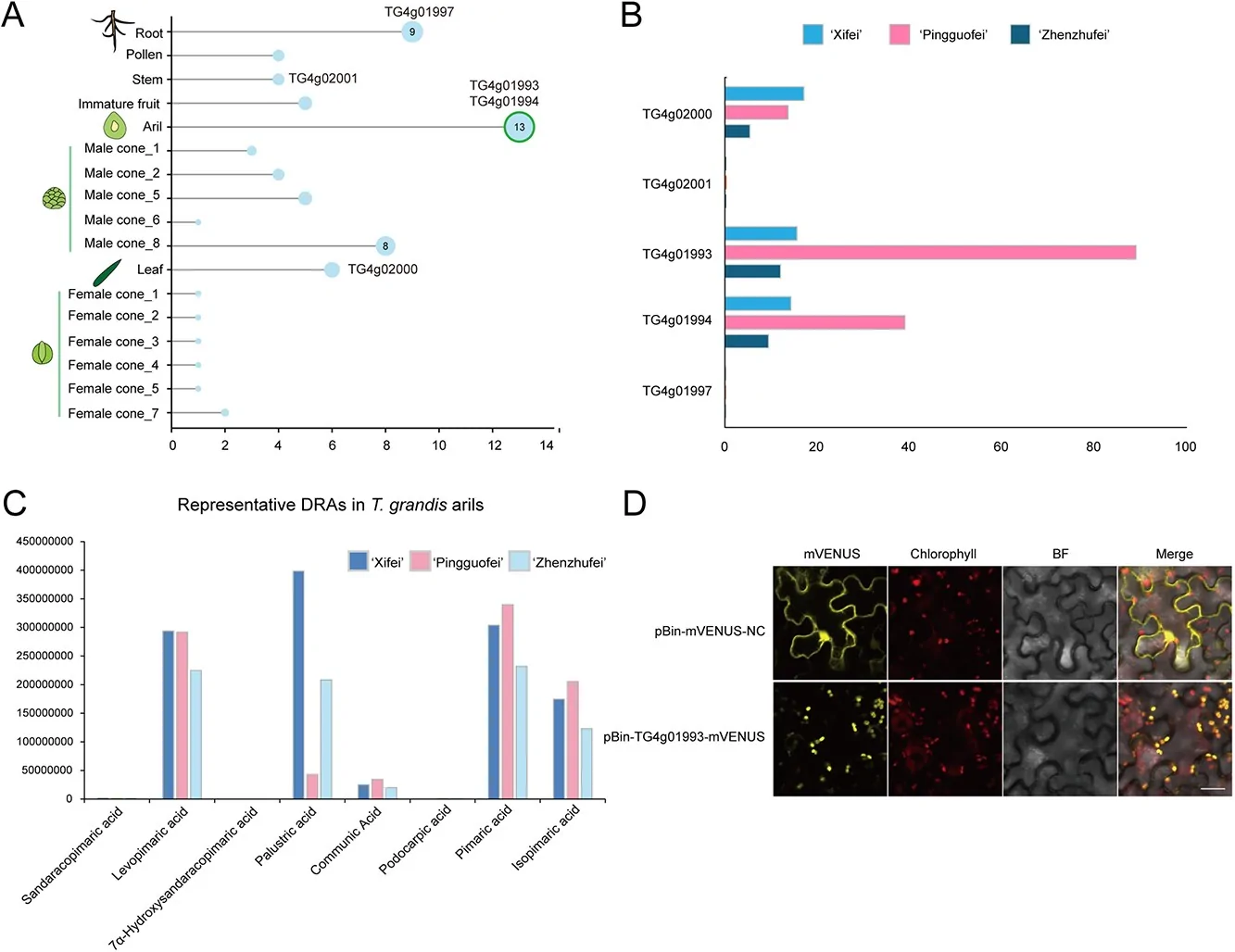
Integrated analysis of terpene synthase (TPS) evolution, tissue-specific gene expression, and terpene accumulation in *Torreya grandis*. (A) Lollipop plot summarizing tissues, which were preferred ones with the highest τ values in *T. grandis*. Genes with maximum TPM < 5 were excluded. (B) Gene expression levels (average TPM of three biological replicates) of LAS/ISO orthologous genes in *T. grandis*. Levopimaradiene/abietadiene synthase, LAS; isopimara-7,15-diene synthase, ISO. (C) Terpenoid metabolite accumulation across arils of three different *T. grandis* varieties. (D) Subcellular localization analysis of TG4g1993.

To illustrate how TD shaped regulatory diversification at the locus level, we examined the LAS/ISO gene cluster in *T. grandis*. Five tandemly duplicated TPS-d3 genes within this syntenic block exhibited pronounced expression divergence across roots, stems, leaves, and arils (Fig. 5A, Figs S11 and S21). Even closely related paralogs showed distinct tissue biases. For example, TG4g2001 was predominantly expressed in stems, whereas TG4g2000 displayed leaf-preferential expression (Fig. 5A, Fig. S21). Among three additional TD-derived paralogs, TG4g1993 and TG4g1997 showed highest expression in arils and roots, respectively, while TG4g1994 exhibited relatively broad expression across multiple tissues (Fig. 5A, Fig. S21).

We further analyzed DRA accumulation in the arils of three *T. grandis* varieties (‘Xifei,’ ‘Pingguofei,’ and ‘Zhenzhufei’). Levopimaric acid, palustric acid, pimaric acid, and isopimaric acid were the predominant DRAs, whereas sandaracopimaric acid, 7α-hydroxysandaracopimaric acid, and podocarpic acid were present at relatively low levels (Fig. 5C). Clear quantitative differences in DRA composition were observed among varieties. ‘Xifei’ accumulated particularly high levels of palustric acid, whereas ‘Pingguofei’ showed increased levels of pimaric and isopimaric acids. In contrast, ‘Zhenzhufei’ generally exhibited intermediate or lower levels of most DRAs (Fig. 5C). Minor DRAs, such as communic acid, were detected at low but comparable levels across all three varieties (Fig. 5C).

Consistent with these metabolic differences, TG4g1993, TG4g1994, and TG4g2000 were consistently highly expressed in arils across all three varieties, whereas TG4g2001 and TG4g1997 showed weak or undetectable expression (Fig. 5B). The observed varietal variation in DRA (pimaric acid, isopimaric acid, and communic acid) accumulation closely matched the differential expression patterns of TG4g1993 and TG4g1994 among *T. grandis* varieties (Fig. 5B). Subcellular localization analysis revealed that TG4g1993 co-localized with chloroplast autofluorescence, consistent with the typical subcellular localization pattern of diterpene synthases involved in DRA biosynthesis (Fig. 5D). Collectively, these results suggest TG4g1993 as a strong candidate gene underlying aril-specific DRA biosynthesis in *T. grandis*.

## Discussion

As the phylogenetic sister group of flowering plants, gymnosperms represent one of the most ancient and extant lineages of seed plants and therefore provide a uniquely informative evolutionary framework for studying the origin and diversification of plant metabolic pathways [41]. A distinguishing feature of gymnosperms terpenoid metabolism is the lineage-specific TPS-d subfamily, whose members collectively generate a diverse array of specialized terpenoid metabolites, including mono-, sesqui-, and diterpenes [14, 17, 25]. These compounds are predominantly accumulated in volatile blends and oleoresin deposits, where they play essential roles in defense against biotic stress [42, 43]. In addition, gymnosperm terpenoids have considerable economic value due to their widespread applications in food, pharmaceutical, and cosmetic industries [19]. Together, these features make gymnosperms an ideal system for investigating the evolution of specialized terpenoid metabolism.

Despite rapid advances in genome sequencing and metabolite profiling that have facilitated the identification of numerous terpenoid biosynthetic genes, research on terpenoid metabolism has remained disproportionately focused on angiosperms, particularly model species such as *A. thaliana*, tomato, and rice [8, 44–47]. To date, genomes of over 30 gymnosperm species spanning seven major families including Pinaceae, Cupressaceae, Ginkgoaceae, and Cycadaceae have been sequenced, with genome sizes ranging from 3.86 to 27.61 Gb [31, 48, 49]. Several genomic surveys of gymnosperm TPS families have been reported [30, 50, 51]. For example, the study in *P. massoniana* genome has characterized resin terpene biosynthetic pathways, TPS phylogeny, and TPS conserved motif architecture [30]. Although it is known that the lineage-specific gene duplication contribute to the expansion of the gymnosperm-specific TPS-d subfamily [17], the evolutionary basis of TPS diversity and functional diversification in gymnosperms remains poorly understood, for example, which individual genes within each subclade produce distinct metabolites in each species, and how neofunctionalization arose during evolution. To address these questions, we assembled a dataset of 10 chromosome-level gymnosperm genomes representing the broadest taxonomic coverage available, with an additional six land plant species included for comparative analyses. By integrating gene copy number variation, microsynteny, AAP, and expression specificity, we proposed a plausible evolutionary scenario for TPS-d genes in gymnosperms (Fig. 6). Recurrent TD events including the lineage-specific ones are inferred to contribute to the generation of paralogs, which subsequently undergo sequence divergence and expression reprogramming, collectively promoting functional and metabolic diversification (Fig. 6).

**Figure 6:**
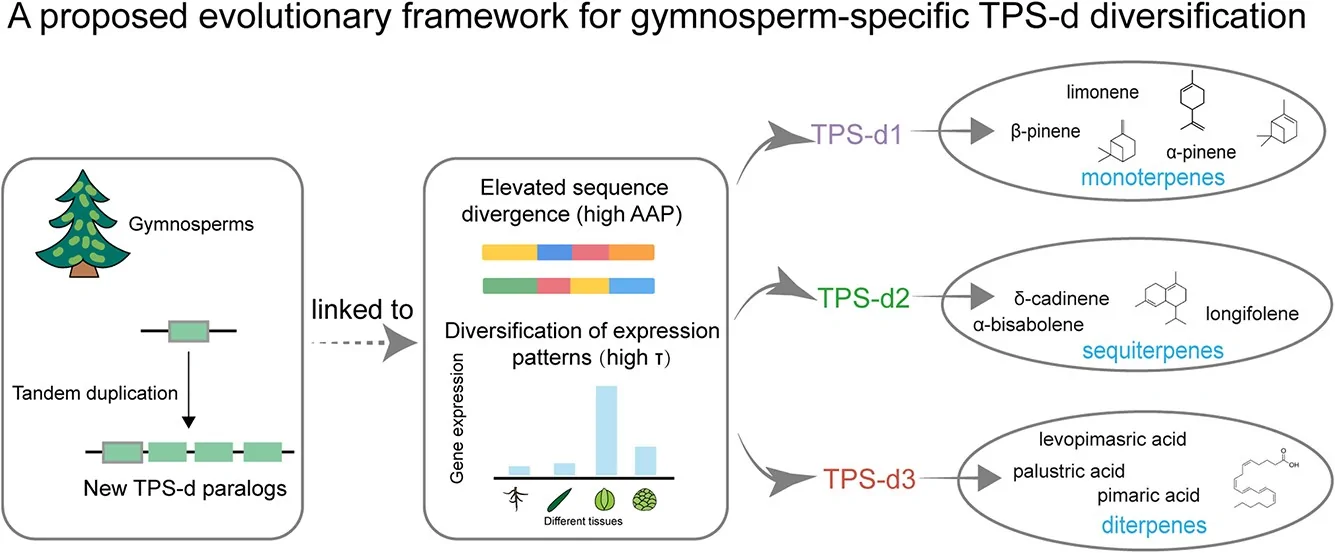
A proposed evolutionary framework illustrating how lineage-specific TD, sequence divergence, and expression diversification collectively shape TPS-d gene evolution in gymnosperms. The main functions of TPS-d subfamilies displayed referred to previous studies [24, 25].

The evolution of typical plant TPS families in higher plants is thought to have involved multiple gene duplication events, followed by subfunctionalization and neofunctionalization, as evidenced by the frequent occurrence of TPS genes in tandem arrays within flowering plant genomes [46, 47, 52]. For example, a sesquiterpene biosynthetic gene cluster *(LcTPSa1*-*LcTPSa4*), consisting of four tandemly duplicated TPS genes was identified in lychee [46]. Although genes located in tandem arrays are not necessarily all derived from recent TDs, the pronounced enrichment of TPS-d genes in tandem clusters, together with the strong lineage-specific expansion patterns observed in phylogenetic analyses, indicated that TD might have been a major contributor to TPS-d gene diversification in gymnosperms.

## Conclusion

Terpenoids constitute one of the most diverse classes of plant specialized metabolites, yet the diversity and evolutionary mechanisms underlying TPS diversification in gymnosperms remain poorly understood. In this study, we addressed this gap by comparative genomics analyses across 16 chromosome-level land plant genomes (including 10 chromosome-level gymnosperm genomes), with a particular focus on the gymnosperm-specific TPS-d subfamily. Our analyses provided a proposed evolutionary framework illustrating how TD, sequence divergence, and expression diversification collectively shaped TPS-d gene evolution in gymnosperms (Fig. 6). We showed that TDs contribute to the expansion of TPS-d genes across gymnosperm lineages. Importantly, these duplicated TPS-d paralogs exhibit accelerated AAP and markedly increased τ values compared with conserved TPS subfamilies involved in primary metabolism. This combination of structural and regulatory divergence indicated rapid functional differentiation following duplication, rather than passive gene dosage effects of TPS-d genes.

## Materials and methods

### Genome data collection and identification of TPSs

The genome assembly and annotation files of 10 gymnosperms, one fern (*A. spinulosa*), two monocots (*A. gramineus* and *O. sativa*), two dicots (*V. vinifera* and *A. thaliana*), and one basal angiosperm (*N. colorata*) were downloaded from Phytozome v12.1.6 (http://phytozome. jgi.doe.gov/pz/portal.html), figshare (https://figshare.com/), CNGBdb (https://db.cngb.org/), Genome Warehouse (https://ngdc.cncb.ac.cn/gwh/Assembly/18742/show), PlantGIR (https://doi.org/10.1093/hr/uhae342) [12], etc. To identify TPS genes, local searches of the downloaded protein sequences of the longest isoforms from the above-mentioned genomes against the Pfam-A database using hmmsearch tool of HMMER [53], with an E-value threshold set to 1e-5 was performed. Sequences were only classified as putative TPS candidates if their top hits matched the two specific HMM profiles: Terpene_synth_C (PF03936) and Terpene_synth (PF01397). Afterwards, protein sequences of the number of amino acids less than 50 were filtered to obtain the final TPS proteomes.

### Species phylogenetic tree construction

The species phylogenetic tree of 16 plant species was constructed by single-copy orthologous genes obtained by Orthofinder [54]. Protein sequences from these single-copy genes were aligned using MAFFT (v 7.467) with default settings [55], followed by extracting conserved blocks using Gblocks [56]. Model test was used to identify the best model for the construction of phylogenetic tree. Subsequent construction of the maximum likelihood tree was performed in RAxML, utilizing the JTT + I + G4 + F substitution model [56]. *A. spinulosa* was designated as the outgroup for tree rooting. The phylogenetic tree was visualized with FigTree (https://tree.bio.ed.ac.uk/software/figtree/).

### Multiple sequence alignment and phylogenetic analysis

Multisequence alignments of the identified TPS protein sequences were performed using MAFFT (v7.310) and were then trimmed by TrimAl (1.4.1) [55, 57]. Phylogenetic trees were inferred using maximum likelihood as implemented in IQTREE (v1.6.12) with best-fit model chosen using Bayesian information criteria [58]. Branch support was tested using 1000 bootstrap replicates. The consensus trees topology was visualized with MEGA [59] and FigTree (http://tree.bio.ed.ac.uk/software/figtree/).

### Transcriptome sequencing and expression analysis

Aril samples were collected from three varieties of *T. grandis*, including ‘Xifei,’ ‘Zhenzhufei’ and ‘Pingguofei’ cultivated under natural field conditions at the Panmugang Resource Garden in Hangzhou, Zhejiang, China in August 2025. For each variety, three biological replicates were prepared, with each replicate consisting of a mixed pool of arils from more than five fruits harvested from a single individual tree.

Total RNA was extracted using Trizol Reagent (Invitrogen). RNA purity and preliminary concentration were assessed using a NanoDrop ND-2000 spectrophotometer (Thermo Fisher Scientific). cDNA library construction was performed following standard procedures, with the detailed workflow as follows: (i) mRNA was enriched from total RNA using Oligo(dT)-attached magnetic beads. (ii) Fragmentation Buffer was added to fragment the enriched mRNA into short segments randomly. (iii) Using the fragmented mRNA as the template, the first-strand cDNA was synthesized with random hexamers. Subsequently, the second-strand cDNA was synthesized by adding reaction buffer, dNTPs, RNase H, and DNA polymerase I. The double-stranded cDNA was then purified using AMPure XP beads. (iv) The purified double-stranded cDNA was subjected to end repair, A-tailing, and ligation of sequencing adapters, followed by fragment size selection using AMPure XP beads. (v) Finally, the target cDNA library was obtained via PCR amplification and enrichment. Upon completion of library construction, library concentration and insert size were assessed using a Qubit 2.0 Fluorometer and an Agilent 2100 Bioanalyzer, respectively. The effective concentration of each library was further accurately quantified by quantitative real-time PCR to ensure strict library quality control. Libraries that passed quality inspection were then used for high-throughput sequencing with a paired-end 150 bp (PE150) read length.

Transcriptome sequencing reads of different species used in our study were retrieved from publicly available RNA-seq datasets in the NCBI (*A. spinulosa*, *C. japonica*, *P. densiflora*, *T. wallichiana*, *V. vinifera*, *T. distichum* var. distichum, *O. sativa*, *N. colorata*, *W. mirabilis*, and *P._tabuliformis*), CNGB (*T. grandis* and *A. gramineus*), and GSA databases (*G. biloba*). The expression matrix of *A. thaliana* was downloaded from the EBI BioStudies database under the accession number E-MTAB-7978 (ftp://ftp.ebi.ac.uk/biostudies/fire/E-MTAB-7978/Files/). Detailed information for these public datasets is provided in the Supplementary Data 2–15. Raw RNA-seq reads were first subjected to quality control using FastQC (v0.11.9) to assess read quality, GC content, and presence of adapter contamination [59]. Clean reads were then aligned to the reference genome using HISAT2 (v2.2.1) [60]. The resulting alignment files were used for transcript assembly and expression quantification with StringTie (v2.2.1) [61]. StringTie was run with default parameters to assemble transcripts and estimate gene expression levels in TPM.

### Calculation of AAP and τ values

Tissue-specific expression profiles of the TPS gene family were quantified using the τ index (ranging from 0 for ubiquitous expression to 1 for tissue-specific expression), which was computed using TBtools software (v. 2.376) [62]. The steps for τ index calculation were: (i) access the ‘TAU Calc’ tool of TBtools software; (ii) import the TPM matrix; (iii) retain default parameters (no manual adjustments); and (iv) execute the analysis module to automatically generate and export τ values for each TPS gene. AAP values of TPS proteins were determined using their protein sequences through the ‘Compute Overall Mean Distance’ function in MEGA, where the p-distance model was applied [59].

### Molecular docking

Molecular docking simulations were performed using AutoDock Vina [63]. Polar hydrogen addition, Gasteiger charge calculation, and rotatable bond assignment for the receptor protein and ligand small molecule were conducted separately in AutoDockTools. The receptor active site was defined as the docking box with an appropriate box size. Docking was run with Vina under default parameters, and the conformation with the lowest binding energy was selected as the optimal result. The optimal complex was visualized using PyMOL: the receptor backbone was displayed in cartoon representation, while the ligand and key amino acid residues were shown in ball-and-stick style with element-based coloring. High-definition images were exported for result analysis.

### Identification and microsyntenic analysis of TPS tandem duplicated gene clusters

Tandem duplicated TPS genes located on the same chromosome, with no more than 10 intervening genes between them, were defined as TD pairs. Microsynteny analysis of TPS-related clusters was carried out by using JCVI [64]. *Ks* values for syntenic gene pairs were computed with TBtools [62], and *Ks* distribution plots were generated using the ggplot2 package in R.

### Estimation of TD event time and species diversification time

The timing of TD and species diversification events was estimated using the formula *T* = *Ks*/2*r*, with a synonymous substitution rate of *r* = 6.5 × 10^−9^ [35]. Additionally, TIMETREE (http://www.timetree.org/home) was used to obtain references for the evolutionary timescale.

### Terpenoid detection of *T. grandis*

Kernels, leaves, arils, male cones, female cones, and tender branches of *T. grandis* cultivated in the Donghu campus of Zhejiang A&F University (Hangzhou, Zhejiang Province, China) were sampled in August for non-targeted metabolomic sequencing, with the experimental method following that described in a previous study [65]. Aril samples (three biological replicates per sample) were collected from ‘Xifei,’ ‘Zhenzhufei,’ and ‘Pingguofei’ grown under natural field conditions at the Panmugang Resource Garden in Hangzhou, Zhejiang Province, China. Wide-targeted metabolomic sequencing was then performed on these samples following the method described by Liang *et al.* [66].

### Subcellular location

Total RNA was extracted from *T. grandis* arils and reverse-transcribed into cDNA. The target gene fragment was amplified by PCR and purified via gel extraction. The pBIN-mVENUS-NC vector was digested with KpnI (Thermo, FD0524), recovered by gel electrophoresis, and fused with the target fragment via homologous recombination (Vazyme, C112-02) to generate the expression construct. The recombinant plasmid was transformed into *E. coli* DH5α, verified by colony PCR and Sanger sequencing, and then introduced into Agrobacterium. Positive Agrobacterium strains were cultured overnight in LB medium (supplemented with Kan and Rif) at 28°C with shaking at 180 rpm. Cells were harvested, washed twice with sterile water, and resuspended in infiltration buffer (100 μl 1 M MgCl_2_, 100 μl 1 M MES, 10 μl 200 mM AS, adjusted to 10 ml) to an OD_600_ of ~0.6, followed by 3 h of dark incubation at 28°C. *Nicotiana benthamiana* leaves were infiltrated with the suspension using a 1 ml needleless syringe, and plants were incubated under low light for 2 d. Leaf discs were mounted on slides, and fluorescence signals of mVENUS (excitation 514 nm / emission 527 nm) and chloroplast autofluorescence (excitation 640 nm) were observed and captured by confocal laser scanning microscopy.

## Supplementary Material

Web_Material_uhag195

## Data Availability

The raw RNA-seq data generated in this study are available in the Zenodo database (https://doi.org/10.5281/zenodo.18886203).
