# Supplementary material for: Tandem duplication underlies diversification of the gymnosperm-specific TPS-d subfamily
Source: Hortic Res. 2026 May 20;13(10):uhag195. doi: 10.1093/hr/uhag195 (PMC13626697; doi:10.1093/hr/uhag195)
Supplement: Supplementary file 1 [file web_material_uhag195.zip › Supplementary information.docx]

Supplementary Information for

**Tandem duplication underlies diversification of the gymnosperm-specific TPS-d subfamily**

Yuwei Liang *et al*.

Corresponding authors: Chengjun Zhang, zhangcj@zafu.edu.cn; Jiasheng Wu, wujs@zafu.edu.cn; Liangsheng Zhang, zls83@zju.edu.cn; Yuwei Liang, yuwei.liang@zju.edu.cn

**The file includes:**

Supplementary Figure 1 to 21

Supplementary Table1 to 4

**Fig. S1. Representative tandem duplicated gene clusters identified in gymnosperms.** (A) TPS-d3 tandem array in *Pinus tabuliformis*. (B) TPS-d1 tandem array in *Torreya grandis*. TPS genes were represented in pink fonts.

**Fig. S2. *Ks* distribution of tandem duplicated TPS genes.**

**Fig. S3. Microsyntenic block of LAS/ISO orthologs between *Pinus densiflora* and *P. tabuliformis*.** Levopimaradiene/abietadiene synthase, LAS; isopimara-7,15-diene synthase, ISO.

**Fig. S4. *Ks* distribution of orthologous gene pairs between *Pinus densiflora* and *P. tabuliformis*.**

**Fig. S5. Statistics of average amino acid polymorphism (AAP) of TPS subfamilies.**

**Fig. S6. Statistics of the amino acid polymorphism of TPS subfamilies in *Nymphaea colorata*.**

**Fig. S7. Statistics of the amino acid polymorphism of TPS subfamilies in representative monocots and dicots.**

**Fig. S8. Statistics of the amino acid polymorphism of TPS subfamilies in representative gymnosperms.**

**Fig. S9. Molecular docking of tandem duplicated LAS/ISO orthologs in *Torrega grandis*.** Levopimaradiene/abietadiene synthase, LAS; isopimara-7,15-diene synthase, ISO.

**Fig. S10. Expression of TPS genes in different tissues and species of selected plant species.** TPS genes exhibiting ubiquitous expression across selected tissues were highlighted with blue boxes, whereas those showing strong tissue specificity were marked with red boxes. Genes with maximum TPM < 5) were considered non-significantly expressed and therefore removed.

**Fig. S11. Accumulation of terpenoids across different tissues in *Torreya grandis*.**

**Fig. S12. Statistics of average tau (τ) values of different TPS subfamilies.** Genes with maximum TPM < 5 excluded.

**Fig. S13. Box plots showing τ values of different TPS subfamilies in *Nymphaea colorata*.** Genes with maximum TPM < 5 excluded.

**Fig. S14. Box plots showing τ values of different TPS subfamilies in *Acorus gramineus* (A)*, Oryza sativa* (B)*, Vitis vinifera* (C)*, and Arabidopsis thaliana* (D).** Genes with maximum TPM < 5 excluded.

**Fig. S15. Box plots showing τ values of different TPS subfamilies in *Pinus densiflora* (A), *Cycas panzhihuaensis* (B), *Ginkgo biloba* (C), and *Welwitschia mirabilis* (D).** Genes with maximum TPM < 5 excluded.

**Fig. S16. Box plots showing τ values of different TPS subfamilies in *Cryptomeria japonica* D. Don (A), *Taxus wallichiana* (B), *Pinus tabuliformis* (C), and *Torreya grandis* (D).** Genes with maximum TPM < 5 excluded.

**Fig. S17. Statistical analysis of mean τ (tau) values of tandem duplicated terpene synthase (TPS) genes representing distinct TPS subfamilies.** Note: Genes with a maximum transcripts per million (TPM) value of less than 5 were excluded from the analysis. The x-axis is arranged sequentially from left to right as follows: tandem duplicated TPS-d genes from seven individual gymnosperm species (*Taxus wallichiana*, *Cryptomeria japonica* D. Don, *Cycas panzhihuaensis*, *Ginkgo biloba*, *Pinus densiflora*, *Torreya grandis*, *Pinus tabuliformis*) and the TPS-d1, TPS-d2, and TPS-d3 subfamilies encompassing all seven species.

**Fig. S18. Lollipop plots summarizing tissues which were preferred ones with the highest τ values in *Nymphaea colorata* (A) and representative monocots and dicots (B)*.*** Genes with maximum TPM < 5 excluded.

**Fig.S19. Lollipop plots summarizing tissues which were preferred ones with the highest τ values in *Cryptomeria japonica* D. Don (A), *Cycas panzhihuaensis* (B), *Ginkgo biloba* (C), and *Pinus densiflora* (D).** Genes with maximum TPM < 5 excluded.

**Fig. S20. Lollipop plots summarizing tissues which were preferred ones with the highest τ values in *Pinus tabuliformis* (A), *Taxus wallichiana* (B), *Torreya grandis* (C), and *Welwitschia mirabilis* (D).** Genes with maximum TPM < 5 excluded.

**Fig. S21. Heatmap illustrating tissue-specific expression profiling of TPS genes and their corresponding τ values.** Tandem duplicated TPS genes were indicated by bold fonts. Genes belonging to different TPS subfamilies were distinguished by distinct colors. Five orthologs of LAS/ISO genes were highlighted in pink boxes. Transcripts per million (TPM) values were normalized by row. Genes with maximum TPM < 5 excluded. Levopimaradiene/abietadiene synthase, LAS; isopimara-7,15-diene synthase, ISO. Arils consist of the majority component of the immature fruits.

**Supplementary Table 1. Statistics of tandem duplicated TPS-d gene members.**

| **Species** | **TD TPS-d1** | **TD TPS-d1/All TPS-d1** | | **TD TPS-d2** | **TD TPS-d2/All TPS-d2** | **TD TPS-d3** | **TD TPS-d3/All TPS-d3** | **All TD TPS-d** | **TD TPS-d/All TPS-d** | **All TPS-d1** | **All TPS-d2** | **All TPS-d3** | **All TPS-d** |
| --- | --- | --- | --- | --- | --- | --- | --- | --- | --- | --- | --- | --- | --- |
| *Pinus tabuliformis* | 29 | | 70.73% | 19 | 95.00% | 28 | 68.29% | 76 | 74.51% | 41 | 20 | 41 | 102 |
| *Ginkgo biloba* | 8 | | 80.00% | 8 | 88.89% | 6 | 60.00% | 22 | 75.86% | 10 | 9 | 10 | 29 |
| *Cycas panzhihuaensis* | 0 | | NA | 0 | NA | 22 | 68.75% | 22 | 68.75% | 0 | 0 | 32 | 32 |
| *Cryptomeria japonica* D. Don | 28 | | 70.00% | 24 | 61.54% | 19 | 65.52% | 71 | 65.74% | 40 | 39 | 29 | 108 |
| *Welwitschia mirabilis* | 0 | | NA | 12 | 50.00% | 0 | NA | 12 | 50.00% | 0 | 24 | 0 | 24 |
| *Taxus wallichiana* | 2 | | 28.57% | 14 | 100.00% | 9 | 81.82% | 25 | 78.13% | 7 | 14 | 11 | 32 |
| *Taxodium distichum* var. *distichum* | 34 | | 89.47% | 14 | 60.87% | 18 | 94.74% | 66 | 82.50% | 38 | 23 | 19 | 80 |
| *Pinus densiflora* | 21 | | 80.77% | 12 | 36.36% | 28 | 73.68% | 61 | 62.89% | 26 | 33 | 38 | 97 |
| *Torreya grandis* | 10 | | 66.67% | 17 | 62.96% | 10 | 83.33% | 37 | 68.52% | 15 | 27 | 12 | 54 |
| *Sequoiadendron giganteum* | 13 | | 41.94% | 18 | 54.55% | 15 | 65.22% | 46 | 52.87% | 31 | 33 | 23 | 87 |
| Average | 15 | | 69.71% | 14 | 62.16% | 16 | 72.09% | 44 | 67.90% | 21 | 22 | 22 | 65 |

Note: tandem duplicated, TD.

**Supplementary Table 2. Statistics of amino acid polymorphism of TPS gene members.**

| **Species** | **TPS gene number** | **Amino acid polymorphism** |
| --- | --- | --- |
| *Acorus gramineus* | 13 | 0.56 |
| *Arabidopsis thaliana* | 44 | 0.60 |
| *Cryptomeria japonica* D. Don | 120 | 0.54 |
| *Cycas panzhihuaensis* | 37 | 0.44 |
| *Ginkgo biloba* | 29 | 0.39 |
| *Nymphaea colorata* | 39 | 0.36 |
| *Oryza sativa* | 39 | 0.60 |
| *Pinus densiflora* | 98 | 0.43 |
| *Pinus tabuliformis* | 105 | 0.45 |
| *Sequoiadendron giganteum* | 98 | 0.54 |
| *Taxodium distichum* var. *distichum* | 91 | 0.55 |
| *Taxus wallichiana* | 35 | 0.48 |
| *Torreya grandis* | 69 | 0.54 |
| *Vitis vinifera* | 69 | 0.50 |
| *Welwitschia mirabilis* | 26 | 0.54 |
| Average | 61 | 0.50 |

**Supplementary Table 3. Statistics of amino acid polymorphism (AAP) of TPS-d gene members generated by tandem duplication.**

| **TPS-d subclade** | **AAP** |
| --- | --- |
| *Pinus tabuliformis* TPS-d1 | 0.29 |
| *Pinus tabuliformis* TPS-d2 | 0.29 |
| *Pinus tabuliformis* TPS-d3 | 0.40 |
| *Ginkgo biloba* TPS-d1 | 0.29 |
| *Ginkgo biloba* TPS-d2 | 0.30 |
| *Ginkgo biloba* TPS-d3 | 0.40 |
| *Cycas panzhihuaensis* TPS-d3 | 0.46 |
| *Cryptomeria japonica* D. Don TPS-d1 | 0.44 |
| *Cryptomeria japonica* D. Don TPS-d2 | 0.43 |
| *Cryptomeria japonica* D. Don TPS-d3 | 0.49 |
| *Welwitschia mirabilis* TPS-d2 | 0.53 |
| *Taxus wallichiana* TPS-d2 | 0.31 |
| *Taxus wallichiana* TPS-d3 | 0.42 |
| *Taxodium distichum* var. *distichum* TPS-d1 | 0.51 |
| *Taxodium distichum* var. *distichum* TPS-d2 | 0.48 |
| *Taxodium distichum* var. *distichum* TPS-d3 | 0.46 |
| *Pinus densiflora* TPS-d1 | 0.31 |
| *Pinus densiflora*TPS-d2 | 0.31 |
| *Pinus densiflora* TPS-d3 | 0.36 |
| *Torreya grandis* TPS-d1 | 0.33 |
| *Torreya grandis* TPS-d2 | 0.55 |
| *Torreya grandis* TPS-d3 | 0.34 |
| *Sequoiadendron giganteum* TPS-d1 | 0.32 |
| *Sequoiadendron giganteum* TPS-d2 | 0.38 |
| *Sequoiadendron giganteum* TPS-d3 | 0.49 |

**Supplementary Table 4. Statistics of τ values of TPS gene members.**

| **Species** | **TPS** | **Average τ**  **(max gene expression ≥ 5)** | **Average τ** |
| --- | --- | --- | --- |
| *Acorus gramineus* | TPS-a | 0.64 | 0.69 |
|  | TPS-b | 0.84 | 0.75 |
|  | TPS-e/f | 0.65 | 0.78 |
|  | TPS-g | 0.89 | 0.96 |
| *Arabidopsis thaliana* | TPS-a | 0.96 | 0.95 |
|  | TPS-b | 0.97 | 0.97 |
|  | TPS-c | NA | 0.89 |
|  | TPS-e/f | 0.79 | 0.79 |
|  | TPS-g | 0.99 | 0.99 |
| *Cryptomeria japonica D. Don* | TPS-c | 0.74 | 0.57 |
|  | TPS-d1 | 0.89 | 0.70 |
|  | TPS-d2 | 0.87 | 0.81 |
|  | TPS-d3 | 0.8 | 0.74 |
|  | TPS-e/f | 0.79 | 0.77 |
| *Cycas panzhihuaensis* | TPS-c | 0.84 | 0.84 |
|  | TPS-d3 | 0.89 | 0.82 |
|  | TPS-e/f | 0.82 | 0.86 |
| *Ginkgo biloba* | TPS-d1 | 0.94 | 0.93 |
|  | TPS-d2 | 0.93 | 0.92 |
|  | TPS-d3 | 0.93 | 0.95 |
| *Nymphaea colorata* | TPS-b | 0.88 | 0.28 |
|  | TPS-e/f | 0.33 | 0.33 |
|  | TPS-g | NA | 0.85 |
|  | TPS-d3 | 1 | 0.87 |
| *Pinus densiflora* | TPS-d2 | 0.89 | 0.80 |
| *Pinus tabuliformis* | TPS-c | 0.99 | 0.99 |
|  | TPS-d1 | 0.83 | 0.75 |
|  | TPS-d1 | 0.78 | 0.85 |
|  | TPS-d2 | 0.77 | 0.81 |
|  | TPS-d3 | 0.76 | 0.80 |
|  | TPS-e/f | 0.74 | 0.74 |
|  | TPS-c | 0.93 | 0.93 |
| *Torreya grandis* | TPS-c | 0.94 | 0.94 |
|  | TPS-d1 | 0.97 | 0.91 |
|  | TPS-d2 | 0.94 | 0.88 |
|  | TPS-d3 | 0.89 | 0.91 |
|  | TPS-e/f | 0.83 | 0.87 |
| *Taxus wallichiana* | TPS-c | NA | 0.77 |
|  | TPS-d1 | 0.81 | 0.79 |
|  | TPS-d2 | 0.83 | 0.78 |
|  | TPS-d3 | 0.59 | 0.57 |
|  | TPS-e/f | 0.53 | 0.53 |
| *Vitis vinifera* | TPS-a | 0.97 | 0.64 |
|  | TPS-b | 1 | 0.64 |
|  | TPS-c | NA | 0.97 |
|  | TPS-e/f | 0.63 | 0.41 |
|  | TPS-g | 0.91 | 0.86 |
| *Welwitschia mirabilis* | TPS-c | 0.72 | 0.72 |
|  | TPS-d2 | 0.88 | 0.86 |
|  | TPS-e/f | 0.36 | 0.36 |
| *Oryza sativa* | TPS-a | 0.98 | 0.96 |
|  | TPS-e/f | 0.94 | 0.94 |
|  | TPS-g | 0.94 | 0.94 |
|  | Average | 0.77 | 0.79 |
